# Evaluating CNC Milling Performance for Machining AISI 316 Stainless Steel with Carbide Cutting Tool Insert

**DOI:** 10.3390/ma15228051

**Published:** 2022-11-15

**Authors:** Azhar Equbal, Md. Asif Equbal, Md. Israr Equbal, Pranav Ravindrannair, Zahid A. Khan, Irfan Anjum Badruddin, Sarfaraz Kamangar, Vineet Tirth, Syed Javed, M. I. Kittur

**Affiliations:** 1 Department of Mechanical Engineering, Faculty of Engineering and Technology, Jamia Millia Islamia, New Delhi 110025, India; 2 Department of Mechanical Engineering, Cambridge Institute of Technology, Ranchi 835213, India; 3 Mechanical Engineering Section, University Polytechnic, Aligarh Muslim University, Aligarh 202001, India; 4 Department of Automobile Engineering, MVSR Engineering College, Hyderabad 501510, India; 5 Mechanical Engineering Department, College of Engineering, King Khalid University, Abha 61421, Saudi Arabia; 6 Department of Mechanical Engineering, Faculty of Engineering, University of Malaya, Kuala Lumpur 50603, Malaysia

**Keywords:** computer numerical control machine, material removal rate, milling, multi-response optimization, surface roughness

## Abstract

The present study investigates the CNC milling performance of the machining of AISI 316 stainless steel using a carbide cutting tool insert. Three critical machining parameters, namely cutting speed (v), feed rate (f) and depth of cut (d), each at three levels, are chosen as input machining parameters. The face-centred central composite design (FCCCD) of the experiment is based on response surface methodology (RSM), and machining performances are measured in terms of material removal rate (MRR) and surface roughness (SR). Analysis of variance, response graphs, and three-dimensional surface plots are used to analyse experimental results. Multi-response optimization using the data envelopment analysis based ranking (DEAR) approach is used to find the ideal configuration of the machining parameters for milling AISI 316 SS. The variables *v* = 220 m/min, *f* = 0.20 mm/rev and *d* = 1.2 mm were obtained as the optimal machine parameter setting. Study reveals that *MRR* is affected dominantly by *d* followed by *v*. For *SR*, *f* is the dominating factor followed by *d*. *SR* is found to be almost unaffected by *v*. Finally, it is important to state that this work made an attempt to successfully machine AISI 316 SS with a carbide cutting tool insert, to investigate the effect of important machining parameters on *MRR* and *SR* and also to optimize the multiple output response using DEAR method.

## 1. Introduction

According to the AISI (American Iron and Steel Institute) system of grading alloy steel, AISI 316 stainless steel (AISI 316 SS) is mostly utilized following AISI 304 [1,2]. A significant alloy with exceptional qualities, AISI 316 SS can be used in demanding and harsh environments [3,4]. Aerospace sector, nuclear plants [5], food and medical industries [6,7], pipes for oil refineries [8] and water filtration systems [8] are just a few industries that frequently employ AISI 316 stainless steel. Because AISI 316 SS is a hard material and requires specialized tools for its machining, it can be difficult to machine. According to the literature, grade 316 is typically vulnerable to work hardening during machining; it is crucial to avoid this [9]. The machining of AISI 316 SS is challenging due to the high adhesive affinity of the chips with the tool insert at high cutting speeds and high thermal loads. Using sharp tools and replacing worn-out tools right away could be one answer. The other option is to machine AISI 316 SS at a lower speed and a greater feed rate. The use of a carbide tool is appropriate given the aforementioned elements, as well as the makeup of AISI 316 steel [10]. Today, CNC (computer numerical control) machining has almost become a necessary tool for manufacturing businesses to reduce vibration during conventional machining [11]. High accuracy and precision are possible with CNC machines that are not possible with traditional manufacturing equipment [12]. Additionally, CNC machining saves production costs and energy usage. Due of the many benefits that CNC machines provide, several researchers have utilized them to examine the machining capabilities of various materials, including AISI 316 SS.

Prajapati and Patel [13] performed CNC turning of austenitic 316 SS using physical vapour deposition (PVD) coated ceramic insert and also carried out multi response optimization to optimize the surface roughness (*SR*) and material removal rate (*MRR*) of AISI 316 SS. They found that effect of the feed rate was more dominant on *SR*, whereas *MRR* was significantly affected by the depth of cut followed by feed rate. Further, they also observed that cutting speed was less significant for both responses. Hernández et al. [14] carried out the milling of AISI 304 steel using the milling cutter of uncoated carbide inserts and studied the effect of machining parameters on *SR* and hardness. They observed that the hardness of AISI 304 steel was the least affected by an increase in cutting speed and feed rate, however, cutting speed significantly affected *SR* and better surface finish was achieved at higher cutting speeds and smaller feed rates. Chandrasheker et al. [15] performed the turning of AISI 316 stainless using diamond tipped tool, since diamond is a super hard material and has many advantages, as compared to tools made with common abrasives. They concluded that surface finish was significantly affected by cutting speed and was followed by cutting fluid. Yasir et al. [16] reported a significant impact of cutting speed (*v*) and feed rate (*f*) on *SR* during the end milling of AISI 316 SS using a tungsten carbide tool. Based on the ANOVA results, they concluded that *f* was the dominant factor, affecting the *SR*, while *v* had negligible impact on *SR*. Their study established the effect of cutting parameters only on the *SR* and did not consider other important performance measures. The impact of tool coating thickness in the pocket milling of austenite 316 steel was investigated by Santhakumar et al. [17] and cycle time, surface quality, tool wear, microhardness and *MRR* were investigated. Results of their study revealed that *d* was the most influential input parameter for the considered performance measures. They also proposed the optimum coating thickness and validated their findings using the confirmation test. Sutar et al. [18] investigated the influence of machining parameters and coolant types (dry and wet) on the *SR* of the end milled AISI 316L SS. They reported that coolant type was the most dominant parameter affecting the *SR*, followed by *v*, *f* and *d*, respectively. They also considered *SR* as the only output response and did not take into consideration other crucial performance measures. Singh et al. [19] also studied the effect of machining parameters on the *SR* of AISI 316 SS. They concluded that *SR* was dominantly affected by *f* and least effected by *v*. Mashinini et al. [20] used wire electrical discharge machining (WEDM) to produce micro-textures on the tool rake face of AISI 316 SS using tungsten carbide tool and studied the effect of machining parameters on *MRR*, *SR* and tool wear rate. They found feed rate (*f)* to be the most important parameter that affected the machining performance of AISI 316 SS. The milling of AISI 316 SS using two different coated inserts (coated insert and MEGA coated insert) was performed by Shelar et al. [21] and the effect of machining parameters on *SR* and *MRR* was studied. The authors concluded that output responses varied for different inserts used during machining. Dambhare et al. [22] performed the dry and wet machining of AISI 316 SS and the impact of machining parameters on tool wear rate (*TWR)* and *SR* was observed. Their results showed that *SR* and *TWR* were lower in the wet environment than the dry environment. In a study conducted by Masek et al. [23], the machinability of AISI 316 SS after processing by laser cladding (LC) of powder and welded arc additive manufacturing (WAAM) was compared with AISI 316 SS samples made by hot rolling process. They observed that the WAAM and LC specimens did not have deformation layers and their inner structure was not the same as the rolled specimen having uniform austenitic structure. In addition, they also found that WAAM samples showed maximum hardness and least hardness was possessed by rolling samples. They finally suggested that WAAM specimens had better machinability at low cutting conditions. Dry, wet and cryogenic turning of AISI 316 SS was performed by Jawade et al. [24] and *SR* was optimized using the grasshopper optimization algorithm. They observed that cryogenic condition displayed the minimum *SR* and concluded that cryogenic processing resulted in improved productivity and better product quality. Kaayank and Kitoy [25] examined the porosity, surface roughness, microhardness and microstructure of selectively laser melted (SLMed) 316L SS samples. They saw a strain-hardened layer on the surface and subsurface of the SLMed item, and that final machining reduced the SR of the 316L SS by up to 88%. They came to the conclusion that the density of porosity on the surface and subsurface is greatly reduced by the finishing machining process. Alfonso et al. [26] turned AISI 316 L steel in order to determine the relationship between the machining parameters and the cutting regime’s parameters. The early progression of wear was significantly impacted by v and f, and they noted that v is the most important parameter controlling the cutting regime.

The AISI 316 SS grade is a tougher material, and it requires specialized tools for machining, according to a review of the literature. Additionally, it has been observed that the AISI 316 grade is more prone to work hardening. Therefore, additional related experiments are needed to evaluate the viability of machining and to support the work hardening statement. Additionally, research has been done to determine how machining parameters affect the machining of AISI 316 SS, according to the literature. However, the majority of research investigations looked at how machining factors affected the surface roughness (SR) of AISI 316 SS. Minimal research has looked at how machining parameters affect MRR and attempted to maximize the MRR when milling AISI 316 SS. More research must be performed to optimize the machining process parameters for a variety of output reactions, including MRR, as it is a highly important response from the standpoint of productivity. Research articles reveal that researchers have studied the effect of machining parameters on output responses; however, the reasoning behind the observed effect has not been properly explained. To broaden the research and to establish fruitful conclusions pertaining to the machining of AIS 316 SS, the authors present an investigation of the CNC milling of AISI 316 stainless steel using a carbide cutting tool insert. The method utilized in this work presents a systematic approach for experimentally examining the impact of critical machining parameters, including cutting speed (v), feed rate (f), and depth of cut (d), on the rate of material removal and surface roughness using a suitable design of experiment technique, namely RSM (response surface methodology) based FCCCD (faced centred central composite design). It also displays the use of appropriate statistical methods (ANOVA) and a straightforward way for optimizing several responses at once, known as the DEAR method. Thus, the present work makes a significant contribution, pertaining to the knowledge domain of AISI 316 SS machining.

## 2. Materials and Method

**Figure 1:**
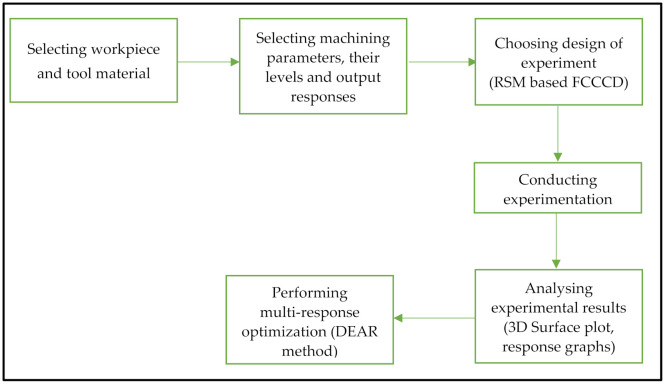
Research Workflow.

Figure 1 shows the different steps that were involved in the present research. In the first stage, workpiece and tool material were selected for conducting the study. After selecting the workpiece and tool material, machining input parameters and output response were decided. Subsequently, experimental investigations were carried out using RSM-based FCCCD, and experimental results were analysed using ANOVA, normality plots, 3D surface plots and response graphs. Multi response optimization was performed using DEAR method to obtain the optimal combinations of machining parameters to optimize *MRR* and *SR*.

### 2.1. Materials

#### 2.1.1. Workpiece Material

AISI 316 is an important austenitic chromium–nickel alloy and standard molybdenum grade, universally known as surgical or marine stainless steel. Compared to AISI 304, it can be used in exceptionally severe and harsh conditions [3]. They are needed where superior corrosion resistance, heat resistance, high toughness, acidic (chloride) resistance and grater tensile strength at elevated temperatures is required. In addition, they possess easy cleaning properties and relatively high strength-to-weight ratio, and they ensure aesthetically pleasing appearance [4]. AISI 316 steel has excellent welding and formability features and can be easily formed into a variety of parts for industrial and architectural applications. Common applications are in (a) aerospace, nuclear plants and the medical industries (b) food processing equipment, especially in chloride environments (c) springs, bolts, screws and nuts (d) oil rig and refinery piping and (e) quarrying and water filtration systems, etc. [27]. Since AISI 316 possesses several crucial properties and has very wide applications, it is selected for investigation in the present study. Chemical composition of workpiece is shown in Table 1. 

#### 2.1.2. Tool Material

Cutting tool material is in the form of cylindrical base made of high speed steel (HSS) to which carbide tool insert is attached. Carbide tools are expensive, and hence they are used as insert and base is taken as HSS.

### 2.2. Data Collection

The values of material removal rate and surface roughness were computed post milling of AISI 316 SS. The methods to calculate the data is as described below:

Material removal rate (*MRR*)—*MRR* was computed in accordance with the formula as given in Equation (1).

(1) $$\mathrm{MRR}=\frac{Volume\;of\;material\;removed\;during\;machining}{Total\;time\;taken}$$

**Figure 2:**
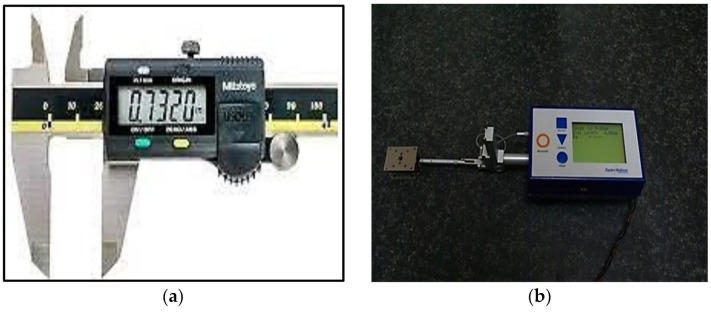
(**a**) Digital Vernier calliper (**b**) Taylor Hobson SURTRONIC 25 machine.

Workpiece dimensions were measured before and after machining using digital Vernier calliper (Figure 2a) (make: Mitutoyo, Country: Delhi, India) of least 0.01 mm and accuracy of +/−0.05 mm. The difference in the volume of the material (mm^3^) before and after machining was then divided with the total machining time (in min.) to obtain the *MRR*. For calculating the material removal, average value of machined dimensions was taken before and after the machining. *MRR* was measured in mm^3^/min.

Surface Roughness (*Ra*)—Taylor Hobson SURTRONIC 25 machine, accuracy of 2% of reading + LSD (least-significant digits) μm (Make: AMETEK, Country: Chennai, India) was used to measure the average *Ra* value (Figure 2b). A probe of diameter 4 mm was calibrated to a reference value of 6 microns using a reference plate. The traverse length was set as 30 mm, and the three measurements were taken along different traverse lines so as not to distort the subsequent reading, due to the probe picking up any abnormality caused by the preceding traverse.

### 2.3. Multi-Response Optimization Methods

Data envelopment analysis based ranking (DEAR) is an integrated approach for multi criteria decision making. It is a relatively simple statistical method applied for optimization of multi-response problems [28,29]. DEAR method is a powerful benchmarking tool and it involves simple computational steps, which can be implemented without using any software for calculation purpose. In this method, there is no need to specify any functional relation between input and output. It is a relatively simple statistical optimization method, where, in addition to comparing decision making unit (DMU) to one another, it may also be used to evaluate a DMU’s progress over time by pooling the data sample [30,31]. In DEAR method, a set of original responses is mapped into a ratio called MRPI (multi-response performance index). MRPI is the ratio between weighted sum of responses with larger the better type and weighted sum of responses with smaller the better or nominal the best type. The optimum level is also affected by the MPRI ratio. The various steps in DEAR method are as follows:

Step 1: Weight determination for each output response via a suitable technique.

Step 2: Data from each output responses are converted into weighted data by multiplying the data with their weight.

Step 3: MRPI is calculated by finding the ratio of weighted data for larger the better type to the smaller the better type or nominal the best type.

Step 4: Determination of optimal levels using maximum MRPI values through analysis of means (ANOM).

## 3. Experimentations

Three crucial machining parameters viz. cutting speed (*v*), feed rate (*f*) and depth of cut (*d*) are chosen as the input variables and material removal rate (*MRR*) and surface roughness (*SR*) are selected as output response. A set of experimentations are designed in accordance with response surface methodology (RSM)-based face centred central composite design (FCCCD), as shown in Table 2. The design matrix consists of three different factors each chosen at three distinct levels and less centres runs, as used by other CCD designs. Parameters and levels of input variables were selected after conducting the pilot experiments. Machining parameters and their levels are presented in Table 3. 

**Figure 3:**
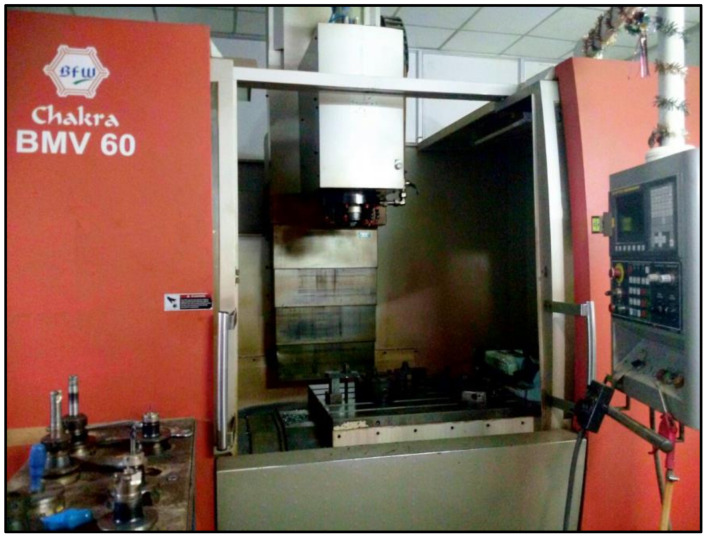
CNC milling machine.

**Figure 4:**
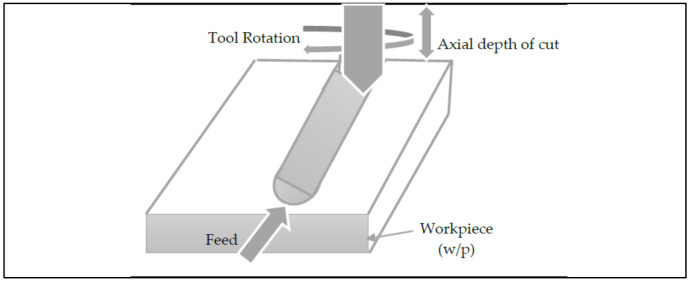
Schematic diagram illustrating the milling process.

CNC milling was then performed following the RSM design, as presented in Table 2, using the machining parameter, as depicted in Table 3. Austenitic AISI 316 stainless steel was selected as the working specimen and carbide cutting tool insert was used. CNC milling was done using CNC machine (Make: BFW, India; Model: Chakra BMV 60), as shown in Figure 3. After machining, data for *MRR* and *SR* of the machined cavities were measured. Digital Vernier calliper and Taylor Hobson SURTRONIC 25 machine, as shown in Figure 2a,b were used to measure *MRR* average *SR* value. The schematic diagram showing the milling process used to cut the slot is presented in Figure 4. In CNC machining, computer control is used to manage the movement and operation of multi-point rotary cutting tools. The tools rotate and move through the surface of the workpiece and slowly remove excess material to achieve the desired dimensions. The CNC milling process basically uses four major steps. In the first stage, 2D or 3D design of the desired part is created. In the second stage, the design is exported into a compatible file format and converted into machine instructions using CAM software. The machine and the workpiece are then prepared. The prepared CNC program is then initiated by the operator and machining starts, in accordance with the given machining instruction. The data collected after experimentations are shown in Table 4. 

## 4. Results and Discussions 

**Figure 5:**
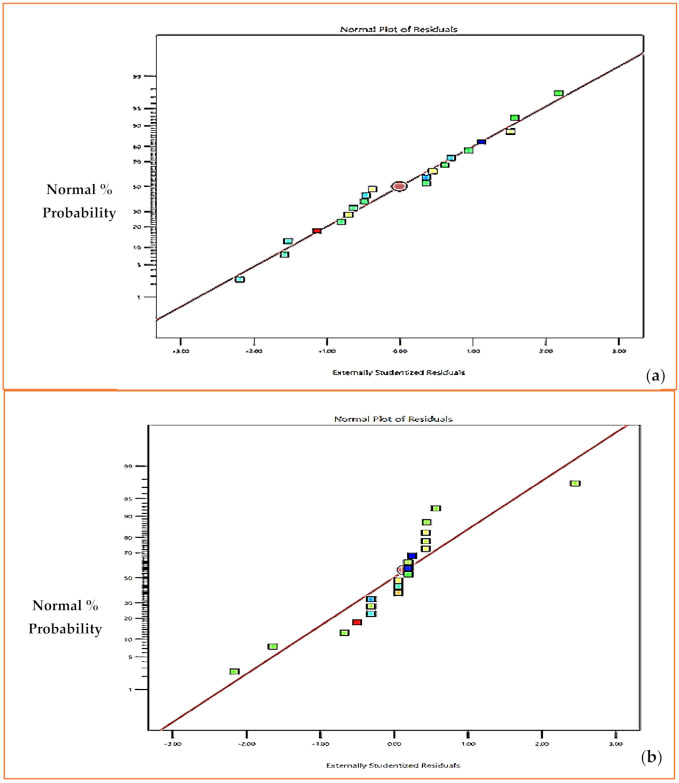
Normality Plot for (**a**) *MRR* (**b**) *SR*.

The significance of parameters and interactions was established from the ANOVA technique using the *p*-value. Here, for a significance level of 5%, *p*-value ≤ 0.05 means that the terms and interactions are significant. The terms and interactions not displaying *p*-value ≤ 0.05 are considered as insignificant. The effectiveness of the developed model was determined using the normality plot, where *p*-value should be ≥0.05. For the normality plot, *p*-value ≥ 0.05 inferred that data are distributed normally. The result of ANOVA for *MRR* and *SR* are presented in Table 5. In Table 5, *SS*, *V* and *DOF* signifies the sum of square, variance and degree of freedom correspondingly; *R*^2^ determines the coefficient of regression and *LOF* represents lack of fit. The parameter *R*^2^ determines the % variation in the machining performance explained by the model. *R*^2^ values are greater than 0.95 for both *MRR* and *SR*, as observed from the results presented in Table 5, which infers that 95% variations in the machining performance are explained by the model developed. It is also apparent from Table 5 that for *MRR*, all three of the machining parameters (*v*, *f* and *d*) are significant, as the *p*-values are less than 0.5 for all. Response surface equations for *MRR* and *SR* are given in Equations (2) and (3). Normality plots for *MRR* and *SR* are presented in Figure 5b; they show that residuals are very near to the line, and thus data are assumed to be normally distributed.

(2) $$MRR=142235.33+3609.25v+2653.45f+7511.49d+553.65vf+553.65vd\;+213.96fd+1156.99v^{2}-1749.95f^{2}+426.74d^{2}$$

(3) $$SR=0.9488+0.0643v+0.2926f+0.1298d+0.0009vf+0.0009vd+0.0009fd\;+0.1023v^{2}-0.2427f^{2}+0.0688d^{2}$$

Since coefficient of regression (*R*^2^) for *MRR* and *SR* is greater than 0.95, respectively, it can be inferred that the models for *MRR* and *SR*, as given in Equations (2) and (3), are good for the prediction of the *MRR* and *SR* results studied in the present research. Response plots and 3D surface graphs are employed for analysing the impact of machining variables on the performance measures. Table 5 shows that for *MRR* and *SR*, all the input parameters, i.e., *v*, *f* and *d*, were dominant or significant. For *MRR*, interactions *v* × *f* and *v* × *d* are significant, but for *SR* no interaction terms are significant. However, to maintain the uniformity, 3D surface plots for all the interactions are shown for explaining the variations in *MRR* and *SR* with changes in input parameters. 

**Figure 6:**
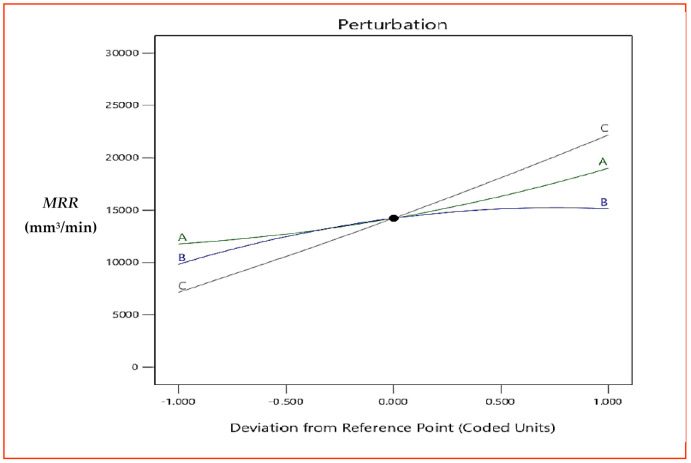
Response graph for *MRR*.

**Figure 7:**
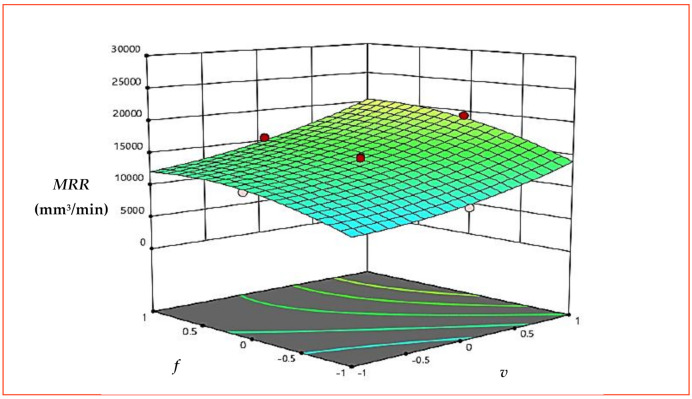
3D surface plot for *MRR* (*v* × *f*).

**Figure 8:**
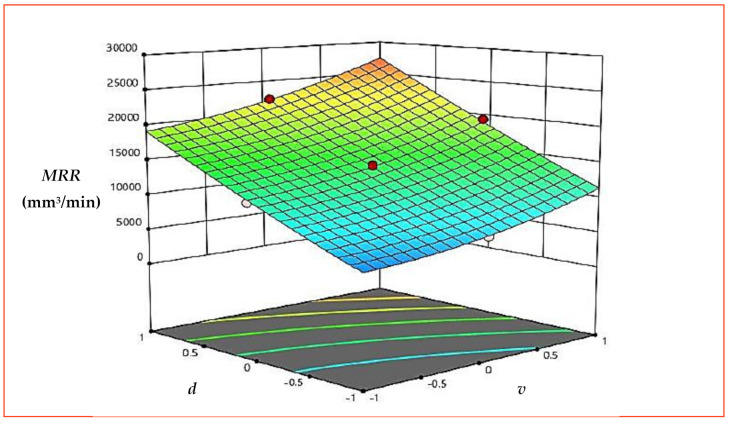
3D surface plot for *MRR* (*v* × *d*).

Figure 6 presents the response plot for *MRR*. Here, A, B and C represent *v*, *f* and *d*, respectively. It is evident from response graph (Figure 6) that with the increase in *v*, *MRR* is expressively affected, and an increase in *MRR* was observed from the start of machining. An increase in cutting speed of workpiece increases the speed of workpiece and time taken for one revolution of workpiece decreases. The speed of revolution of workpiece increases further with increases in cutting speed and more contact timing, and relative motion between workpiece and tool was established, which results in higher *MRR* [32]. Feed is given to the tool and is defined as the advancement of the tool with one rotation of the spindle. With the increase in *f*, *MRR* increases, but the increase is not particularly significant, as compared to the increase in *MRR* observed with *v* and *d*. The increase in *f* will make the tool pass through faster, which could have results in faster *MRR* at the same time, but feed rate used in the research is in mm/rev and increase in *f* is not substantial enough to remove as much material, as observed with *v*. With the increase in *d*, *MRR* increases significantly from the beginning of machining. An increase in *d* removes more material with each cycle of machining. Moreover, an increase in *d* was in combination with an increase in *v*, which means more thickness of material is removed and more relative motion was established between workpiece and tool, resulting in significant *MRR*. The percentage contribution, as presented in the ANOM table (Table 5) by the individual factors (*v*, *f* and *d*), also showed that *MRR* is significantly affected by *d* (72%), followed by *v* (17%) and *f* (9%). The 3D surface plots presented in Figure 7 and Figure 8 draw the same conclusions. 

**Figure 9:**
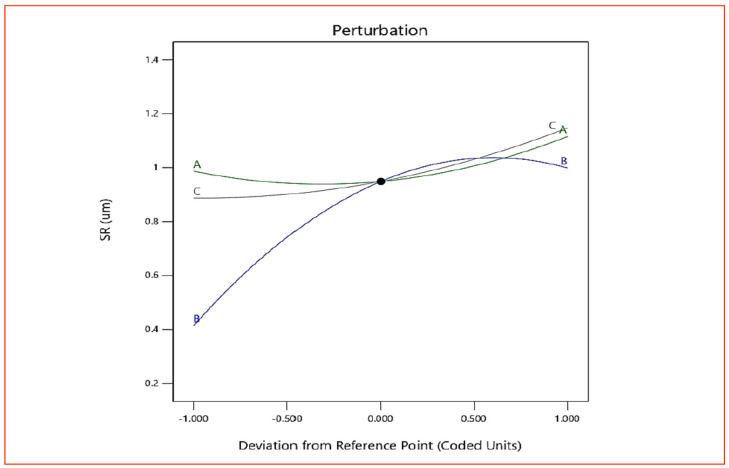
Response graph for *SR*.

**Figure 10:**
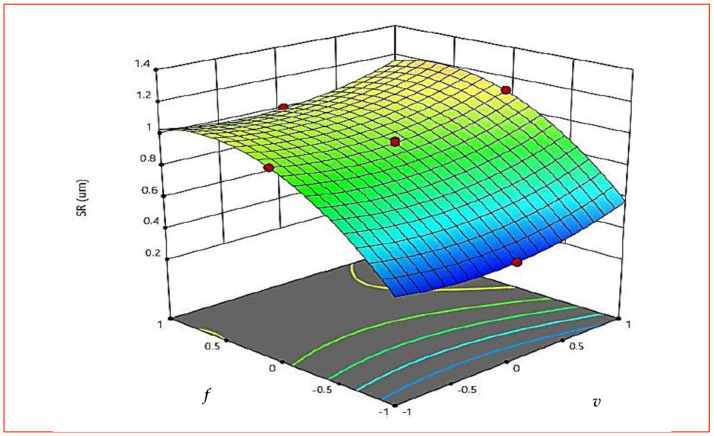
The 3D surface plot for *SR* (*v* × *f*).

**Figure 11:**
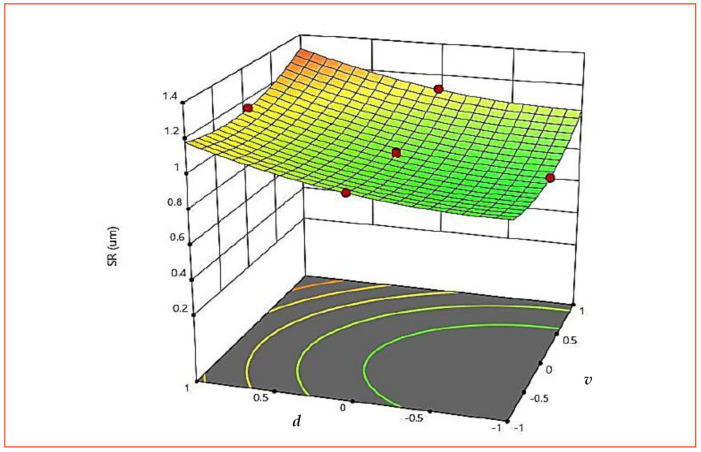
The 3D surface plot for *SR* (*v* × *d*).

As per Table 5, all three machining parameters (*v*, *f* and *d*) are significant but none of the interaction terms is significant for *SR*. However, to correlate the effect of the machining parameter on *SR*, variations for all the interactions are explained. Figure 9 presents the response graph for *SR*. Figure 9 also uses A, B and C to represent *v*, *f* and *d*, respectively. The *SR* varies nonlinearly with the increase in cutting speed (*v*) at the beginning. The roughness reduces during the first phase in the machining to a considerable amount and then increases. With initial increase in *v* built-up-edge formation decreases, which reduces the roughness value. In addition, higher *v* increases the temperature of the cutting zone and material becomes soft, and hence complete removal of material is easier by the cutting tool, which eventually decreases *SR*. A decrease in *SR* with the initial increase in *v* and then its increase at higher *v* proved that *v* can be regarded as a critical cutting velocity in the machining of AISI 316 SS [16]. The effect of *f* on *SR* showed that with an increase in *f*, more thrust forces and chatter are produced during machining, which act on the surface and also produce vibrations, leading to increased *SR* [16]. At lower *d*, less *MRR* was observed and also less heat is produced by friction at the chip–tool interface, resulting in a lesser increase in *SR*. However, at the increased *d*, induced friction at the tool–work interface is higher and substantial material is removed, which increases the chance of unevenness in the surface, thereby increasing the value of *SR*. Figure 10 and Figure 11 (3D surface plot) draws the same conclusion, as observed with the graph in Figure 9.

A comparison of the findings of the present study with the previous related work reveals that, like the earlier research, *MRR* is dominantly affected by the depth of cut (*d*), as the value of *d* is more the *MRR* is also more. Similar to previous research, surface roughness is found to be significantly affected by *f*. It is also observed that *v* has the least significant effect on both *MRR* and *SR*.

## 5. Multi-Response Optimization

**Figure 12:**
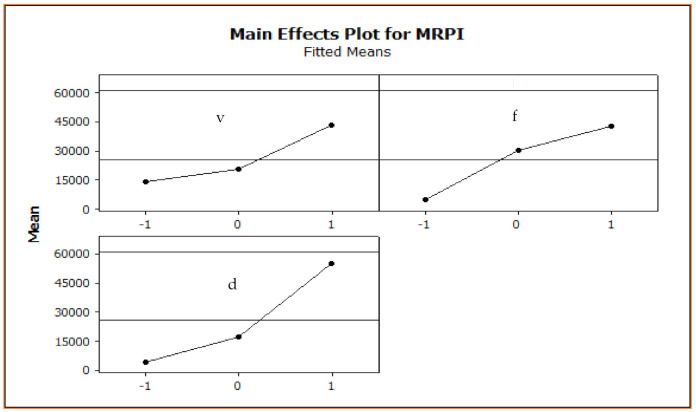
Main effect plot for MRPI.

Data envelopment analysis based ranking (DEAR) method is applied for the optimization of machining parameters. The results of optimization are displayed in Table 6. For *MRR*, it was considered to be the larger the better, and for *SR*, it was considered to be the smaller the better. For the determination of weights for *MRR* (*W_MRR_*), the ratio between individual *MRR* data and the total sum of *MRR* value is considered. Reverse normalization procedure is used in the case of *SR*. Here, for each *SR* data, 1/*SR* is found and then *W_SR_* is calculated. Finally, MRPI is calculated. Once the MRPI is calculated, the analysis of means (ANOM) is applied and optimum machining parameter is determined. The graph of ANOM is shown in Figure 12. The optimum machining parameter obtained using the graph is [1, 1, 1] in coded units and the actual values are *v* = 220 m/min, *f* = 0.20 mm/rev and *d* = 1.20 mm.

## 6. Conclusions

In the present study, an investigation on the effect of three important machining parameters, i.e., cutting speed, feed rate and depth of cut on two output responses viz. material removal rate and surface roughness during the machining of AISI 316 SS, on a CNC milling machine using carbide cutting tool insert was carried out. The study established that CNC milling is an efficient machining process for the machining of AISI 316 SS. It was observed that *MRR* was affected significantly by *d,* followed by *v*. *d* in combination with increased *v* removed more thickness of material and since relative motion and contact established between workpiece and tool was higher, it resulted in significant *MRR*. Increase in cutting speed (*v*) resulted in higher *MRR*, as time taken for the revolutions of workpiece decreased and more contact time and relative motion between workpiece and tool was established. An increase in *f* allowed the tool to travel faster, which resulted in faster *MRR*, but an increase in *f* was not substantial enough to remove high material. Percentage contribution, as shown in Table 5, reveals that *MRR* is only affected by 9.10% with variation in *f*. For *SR*, *f* was found to be the dominating machining parameter, followed by *d*. The effect of *f* on *SR* showed that with an increase in *f*, more thrust forces and chatter are produced during machining, which act on the surface and also produce vibrations, leading to increased *SR*. At higher *d*, induced friction at the tool–work interface is higher and even more material is removed, which increases the chance of unevenness in the surface, thereby increasing the value of *SR*. Decrease in *SR* with the initial increase in *v* and then its increase at higher *v* shows that there is a critical value of *v*, at which stage the machining of AISI 316 SS should be done. Optimization results by data envelopment analysis based ranking method showed that optimal parameters setting for multi-response optimization is *v* = 220 m/min; *f* = 0.20 mm/rev and *d* = 1.2 mm. 

Finally, it should be stated that the methodology adopted in this work presented a systematic approach for experimentally investigating the effect of important machining parameters viz. speed (*v*), feed rate (*f*) and depth of cut (*d*) on the material removal rate (*MRR*) and surface roughness (*SR*) during the machining of AISI 316 SS using a suitable design of the experiment method, i.e., RSM-based FCCCD. In addition, it also demonstrated the use of suitable statistical techniques (ANOVA, response graphs and 3D surface plots) for the analysis of experimental results and the application of a relatively simple multi response optimization method, i.e., DEAR method for simultaneous optimization of multiple responses. However, within the given constraints, the present study made a sincere effort to provide useful information pertaining to the machining of a widely used relatively harder material, i.e., AISI 316 SS; however, to analyse its machining deeply, the effects of other parameters, such as tool material, tool geometry and coolant, etc., may be investigated in future research.

## 7. Limitation and Future Scope

The machining of AISI 316 SS was done with the help of carbide tipped insert. The effect of three critical machining parameters (*v*, *f* and *d*) on *MRR* and *SR* was explored. The effect of other parameters, such as tool material, tool geometry and coolant, etc., on several output responses, such as cutting forces, power, chip morphology, surface integrity, etc., may be explored in future studies to determine which parameters significantly affect the output responses. In addition, the effect of machining parameters on microstructures and mechanical properties may also be investigated by researchers in the future. Future studies may also use other multi criteria decision making (MCDM) methods, fuzzy-based MCDM methods and evolutionary optimization methods, such as PSO, SA, TLBO, NSGA II, etc., for multi-response optimization. 

## Figures and Tables

**Table 1:** Chemical composition of workpiece used.

Element	C	Mn	N	Si	P	S	Cr	Mo	Ni
wt.%	0.08	2.0	0.1	0.75	0.045	0.03	16–18	2–3	10–14

**Table 2:** Experimental matrix obtained from RSM-based FCCCD design.

Exp. No.	Machining Parameter (Coded)		
	*v*	*f*	*d*
1	−1	−1	−1
2	1	−1	−1
3	−1	1	−1
4	1	1	−1
5	−1	−1	1
6	1	−1	1
7	−1	1	1
8	1	1	1
9	−1	0	0
10	1	0	0
11	0	−1	0
12	0	1	0
13	0	0	−1
14	0	0	1
15	0	0	0
16	0	0	0
17	0	0	0
18	0	0	0
19	0	0	0
20	0	0	0

**Table 3:** Machining parameters and their levels.

Machining Parameter	Symbol	Levels			Unit
		1	2	3	
		Low Level(−1)	Centre Level (0)	High Level (+1)	
Cutting speed	*v*	110	165	220	m/min
Feed rate	*f*	0.10	0.15	0.20	mm/rev
Depth of cut	*d*	0.4	0.8	1.20	mm

**Table 4:** Experimental Data.

S. No.	*v*	*F*	*D*	*MRR* (mm^3^/min)	*SR* (µm)
1	1	−1	−1	6733.82	0.517114
2	−1	−1	−1	1790.86	0.39444
3	0	0	0	14,544.3	0.96444
4	1	1	1	28,990	1.36477
5	1	0	0	19,354.3	1.11810
6	−1	1	1	19,617.8	1.23477
7	0	0	0	13,970.3	0.93700
8	1	−1	1	22,261.9	0.778105
9	0	0	1	22,290.7	1.15009
10	1	1	−1	12,374.9	1.10378
11	−1	0	0	11,429.8	0.98810
12	0	0	0	14,444.1	0.95342
13	0	1	0	15,597.8	1.00144
14	−1	−1	1	15,335.5	0.648105
15	0	0	0	14,072.4	0.94344
16	0	0	0	14,023.1	0.93444
17	0	−1	0	9372.42	0.414771
18	0	0	0	14,358.8	0.952429
19	−1	1	−1	5448.5	0.973781
20	0	0	−1	7032.9	0.889095

**Table 5:** ANOVA results for *MRR* and *SR*.

Source	*DOF*	*MRR*						*SR*			
		*SS*	*MS*	*F*	*p*-Value	% Contribution	*SS*	*MS*	*F*	% Contribution	*p*-Value
*v*	1	1.303 × 10^8^	1.303 × 10^8^	1089.11	<0.0001	16.69	0.0413	0.0413	599.63	3.36	<0.0001
*f*	1	7.041 × 10^7^	7.041 × 10^7^	588.65	<0.0001	9.10	0.8562	0.8562	12,429.77	69.61	<0.0001
*d*	1	5.642 × 10^8^	5.642 × 10^8^	4717.25	<0.0001	72.28	0.1684	0.1684	2444.64	13.69	<0.0001
*v* × *f*	1	2.452 × 10^6^	2.452 × 10^6^	20.50	0.0011	0.31	6.707 × 10^−6^	6.707 × 10^−6^	0.0974	0.00	0.7614
*v* × *d*	1	2.452 × 10^6^	2.452 × 10^6^	20.50	0.0011	0.31	6.711 × 10^−6^	6.711 × 10^−6^	0.0974	0.00	0.7614
*f* × *d*	1	3.662 × 10^5^	3.662 × 10^5^	3.06	0.1107	0.047	6.703 × 10^−6^	6.703 × 10^−6^	0.0973	0.00	0.7615
*v* × *v*	1	3.681 × 10^6^	3.681 × 10^6^	30.78	0.0002	0.47	0.0288	0.0288	418.18	2.34	<0.0001
*f* × *f*	1	8.421 × 10^6^	8.421 × 10^6^	70.41	<0.0001	1.08	0.1619	0.1619	2350.82	13.16	<0.0001
*d* × *d*	1	5.008 × 10^5^	5.008 × 10^5^	4.19	0.0679	0.064	0.0130	0.0130	189.16	1.06	<0.0001
Residual	10	1.196 × 10^6^	1.196 × 10^5^				0.0007	0.0001			
*LOF*	5	9.000 × 10^5^	1.800 × 10^5^	3.04	0.1239		0.0000	9.039 × 10^−6^	0.0702		0.9944
Error	5	2.961 × 10^5^	59,224.14				0.0006	0.0001			
Total	19	7.806 × 10^8^					1.23				
		*R^2^* = 0.99						*R^2^* = 0.99			

**Table 6:** Results of DEAR Method.

Exp. No.	*MRR*	*W_MRR_*	*SR*	*1/SR*	*W_SR_*	*MRR × W_MRR_*	*SR × W_SR_*	MRPI
1	6733.82	0.024	0.517	1.934	0.079	160.202	0.153	1045.957
2	1790.86	0.006	0.394	2.535	0.104	11.331	0.263	43.043
3	14,544.3	0.051	0.964	1.037	0.042	747.363	0.044	16,972.827
4	28,990	0.102	1.365	0.733	0.03	2969.219	0.022	135,030.993
5	19,354.3	0.068	1.118	0.894	0.037	1323.429	0.033	40,395.625
6	19,617.83	0.069	1.235	0.81	0.033	1359.714	0.027	50,616.507
7	13,970.3	0.049	0.937	1.067	0.044	689.536	0.047	14,781.168
8	22,261.9	0.079	0.778	1.285	0.053	1750.936	0.068	25,883.259
9	22,290.7	0.079	1.15	0.869	0.036	1755.469	0.031	56,692.964
10	12,374.87	0.044	1.104	0.906	0.037	541.037	0.034	16,094.028
11	11,429.8	0.04	0.988	1.012	0.041	461.555	0.042	11,002.758
12	14,444.1	0.051	0.953	1.049	0.043	737.101	0.045	16,359.408
13	15,597.8	0.055	1.001	0.999	0.041	859.553	0.041	21,047.216
14	15,335.5	0.054	0.648	1.543	0.063	830.886	0.098	8521.278
15	14,072.4	0.05	0.943	1.06	0.043	699.652	0.046	15,204.88
16	14,023.08	0.05	0.934	1.07	0.044	694.756	0.047	14,811.797
17	9372.42	0.033	0.415	2.411	0.099	310.348	0.238	1303.582
18	14,358.8	0.051	0.952	1.05	0.043	728.42	0.045	16,133.166
19	5448.5	0.019	0.974	1.027	0.042	104.882	0.043	2428.257
20	7032.9	0.025	0.889	1.125	0.046	174.749	0.052	3372.744

## Data Availability

Data is available in paper itself.
